# Annexin A8 is a novel molecular marker for detecting lymph node metastasis in oral squamous cell carcinoma

**DOI:** 10.18632/oncotarget.6639

**Published:** 2015-12-17

**Authors:** Ryota Oka, Koh-ichi Nakashiro, Hiroyuki Goda, Kazuki Iwamoto, Norihiko Tokuzen, Hiroyuki Hamakawa

**Affiliations:** ^1^ Department of Oral and Maxillofacial Surgery, Ehime University Graduate School of Medicine, Toon, Ehime 791-0295, Japan

**Keywords:** oral squamous cell carcinoma (OSCC), lymph node metastasis, keratin 19 (KRT19), annexin A8 (ANXA8), reverse transcription loop-mediated isothermal amplification (RT-LAMP)

## Abstract

Cervical lymph node metastasis is an important prognostic factor in oral squamous cell carcinoma (OSCC), but its accurate assessment after sentinel node biopsy or neck dissection is often limited to the histopathological examination of only one or two sections. Previous our study showed the usefulness of the reverse transcription loop-mediated isothermal amplification (RT-LAMP) targeting keratin 19 (KRT19) mRNA for the genetic detection of lymph node metastasis, but the sensitivity was insufficient. Here, we have attempted to identify novel molecular markers for OSCC cells in lymph nodes. We performed microarray analysis to identify genes overexpressed in 7 metastatic lymph nodes from OSCC patients, compared to 1 normal lymph node and 5 salivary glands from non-cancer patients. We then used real-time quantitative RT-PCR (qRT-PCR) and RT-LAMP to compare the expression of these genes in newly resected metastatic and normal lymph nodes. Of 4 genes identified by microarray analysis, annexin A8 (ANXA8) and desmoglein 3 mRNA were detected by qRT-PCR in metastatic lymph nodes but not in normal lymph nodes. Furthermore, ANXA8 mRNA expression was detected in all KRT19-negative metastatic lymph nodes. Both KRT19 and ANXA8 mRNA may be useful markers for detecting lymph node metastases in OSCC patients.

## INTRODUCTION

An estimated 263,900 new cases and 128,000 deaths from oral cavity cancer occurred in 2008 worldwide [1]. The prognosis for oral squamous cell carcinoma (OSCC) patients is determined by multiple factors including primary tumor size (T), the presence of lymph node (N) and distant (M) metastases, and histopathological grade [2, 3]. Of these, N is of paramount importance [4-6], so it is important to precisely diagnose the metastatic status of lymph nodes. Ultrasound (US), computed tomography (CT), magnetic resonance imaging (MRI), and positron emission tomography (PET)-CT have all been used to detect cervical lymph node metastasis. Individually, the sensitivity of these methods is approximately 70-80% [7, 8], while in combination this improves to 86.5% [8]. However, micrometastases less than 2 mm in diameter are difficult to detect by these imaging techniques, so when we diagnose OSCC patients as clinically N0 (cN0), we may be failing to detect micrometastasis in lymph nodes. Elective neck dissection for OSCC patients with cN0 is a standard [9], but may be over-treatment in approximately 80% of these patients [10]. Introducing sentinel node biopsy (SNB) in cN0 patients could both avoid unnecessary neck dissections and be more financially economical [11, 12], but the limitation to diagnosis then becomes our ability to histopathologically detect micrometastases. Currently this depends upon the examination of the largest surface area on one or two sections of the resected sentinel lymph nodes and to detect micrometastases in only a few slides is often difficult. Thus, a method based on molecular biology that uses whole lymph nodes may provide a more accurate diagnosis than conventional histopathology.

Recently, we have used an automated system that rapidly quantifies keratin 19 (KRT19) mRNA using the reverse transcription loop-mediated isothermal amplification (RT-LAMP) method to examine sentinel lymph nodes from OSCC patients assessed as cN0. This method is superior to PCR in its simplicity, rapidity, specificity, and cost-effectiveness [13]. At present, the RT-LAMP for KRT19 is used clinically for SNB in breast, colon, and gastric cancer patients [14-16]. In a previous study, we showed that the sensitivity and specificity of this assay in OSCC patients were 86.9% and 97.2%, respectively [17]. The sensitivity of the RT-LAMP for KRT19 in OSCC patients was lower than has been reported in other cancers. This difference in sensitivity may be due to differences in the relative expression levels of KRT19 in different cancers. In several primary OSCC tumors and associated metastatic lymph nodes, KRT19 mRNA and protein were expressed at extremely low levels [17]. The sensitivity of the RT-LAMP could be improved by using multiple markers. Therefore, here we attempted to identify more efficient molecular markers for detecting lymph node metastasis in OSCC patients.

## RESULTS

### Identification of overexpressed genes in metastatic lymph nodes from OSCC patients

**Figure 1:**
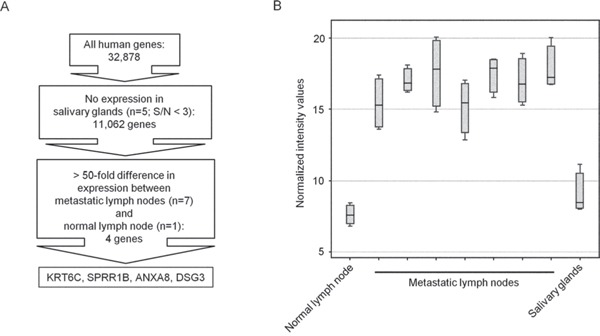
Identification of candidate molecular markers for detecting lymph node metastases in OSCC by microarray analysis **A.** Experimental plan for microarray analysis. **B.** Box plot of keratin 6C (KRT6C), small proline-rich protein 1B (SPRR1B), annexin A8 (ANXA8), and desmoglein 3 (DSG3) mRNA expression levels in a normal lymph node, metastatic lymph nodes, and salivary glands.

Using microarray analysis, we compared the gene expression profiles in 7 metastatic lymph nodes from OSCC patients and 1 cervical lymph node from a non-cancer patient. We also analyzed 5 salivary glands, because heterotopic salivary gland tissue may also be present in cervical lymph nodes [18]. We evaluated gene expression in terms of signal to noise ratios, with a ratio under 3 taken as negative expression and a ratio over 3 taken as positive expression. We identified 11,062 genes which were not expressed in any of the salivary glands tested. Amongst these genes, we found 4 that were overexpressed in all metastatic lymph nodes by more than 50 times compared to the normal lymph node (Figure 1A, 1B). These genes were keratin 6C (KRT6C), small proline-rich protein 1B (SPRR1B), annexin A8 (ANXA8), and desmoglein 3 (DSG3).

### Overexpression of ANXA8 and DSG3 in metastatic lymph nodes by quantitative RT-PCR (qRT-PCR)

**Figure 2:**
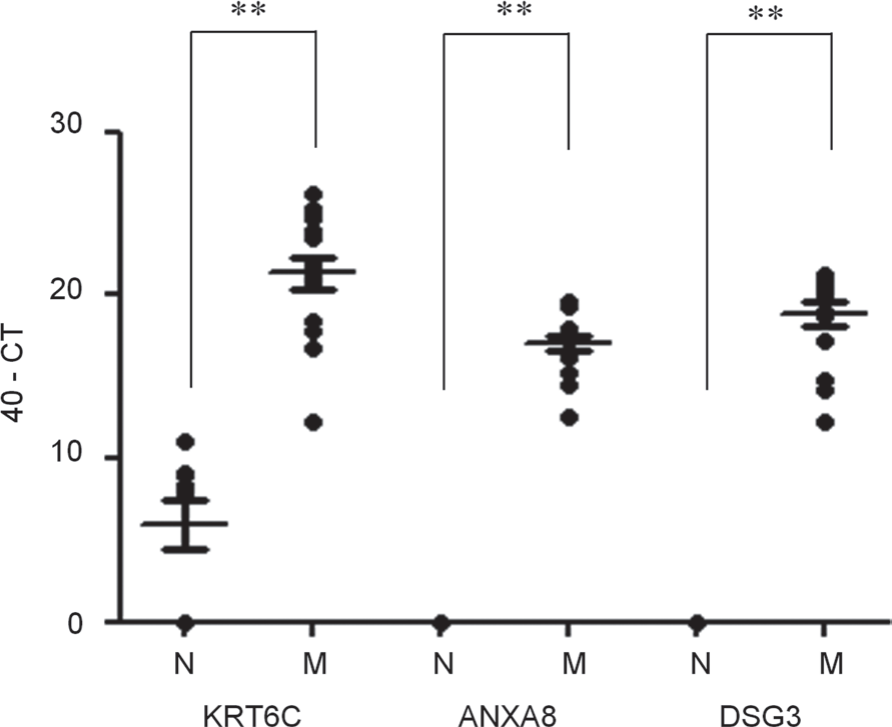
Quantification of candidate molecular markers in normal and metastatic lymph nodes by qRT-PCR Three genes, KRT6C, ANXA8, and DSG3, were evaluated by qRT-PCR using 23 metastatic lymph nodes (M) and 9 normal lymph nodes (N), as defined histopathologically. CT, cycle threshold; **, *p* < 0.0001 compared to normal lymph nodes.

We were able to design primers for RT-PCR for KRT6C, ANXA8, and DSG3 but not for SPRR1B. The expression of these 3 genes was examined by qRT-PCR in 23 fresh metastatic lymph nodes from OSCC patients and 9 normal lymph nodes from non-cancer patients. All 3 genes were significantly overexpressed in the metastatic lymph nodes compared to the normal lymph nodes. In particular, neither ANXA8 nor DSG3 mRNA was detected in any of the normal lymph nodes (Figure 2).

### Expression of ANXA8 and DSG3 in KRT19 negative lymph nodes by RT-LAMP

**Figure 3:**
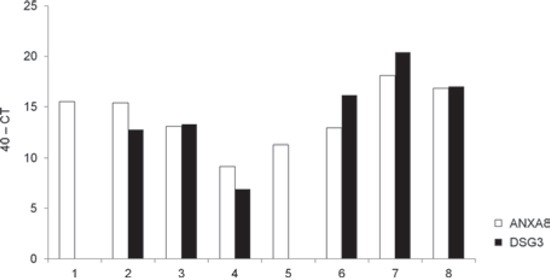
Expression of ANXA8 and DSG3 mRNA in KRT19-negative metastatic lymph nodes The expression of ANXA8 (open bars) and DSG3 (solid bars) mRNA was evaluated by qRT-PCR in 8 lymph nodes (1-8) that had been identified as negative for KRT19 mRNA expression in RT-LAMP assays. CT, cycle threshold.

In a previous study, we found that RT-LAMP could detect KRT19 mRNA in 53 of 61 (86.9%) metastatic lymph nodes from OSCC patients [17]. Using qRT-PCR, we asked whether the expression of ANXA8 and DSG3 mRNAs could be detected in the 8 metastatic lymph nodes in which RT-LAMP had been unable to detect KRT19 expression. We were able to detect DSG3 mRNA in 6 of the 8 lymph nodes, whereas ANXA8 mRNA was detected in all 8 lymph nodes (Figure 3).

### Detection rates in metastatic lymph nodes using single or double markers

We investigated detection rates using a single marker (KRT19 or ANXA8) or double markers (KRT19 and ANXA8), using RT-LAMP to detect KRT19 mRNA and qRT-PCR to detect ANXA8 mRNA. Among 58 lymph nodes classified histopathologically as metastasis-positive, KRT19 mRNA was detected in 86.2% (50/58) and ANXA8 mRNA was detected in 87.9% (51/58). When we used both markers (KRT19 and ANXA8), we were able to detect expression in all metastatic lymph nodes (Table 1). We also tested 253 lymph nodes classified histopathologically as metastasis-negative and found that the qRT-PCR assay detected ANXA8 mRNA expression in approximately 3% of the samples (Table 1).

**Table 1:** Detection rate of KRT19 mRNA by RT-LAMP and ANXA8 mRNA by qRT-PCR in lymph nodes classified as positive or negative for metastases histopathologically, using single or double markers

	KRT19	ANXA8	KRT19 + ANXA8
Histopathologically positive lymph nodes (n=58)	50 (6)/58 (7) 86.2%	51 (5)/58 (7) 87.9%	58 (7)/58 (7) 100%
Histopathologically negative lymph nodes (n=253)	0 (0)/253 (65) 0%	7 (2)/253 (65) 2.8%	7 (2)/253 (65) 2.8%

( ), Number of sentinel lymph nodes

### Detection of ANXA8 expression by RT-LAMP

**Figure 4:**
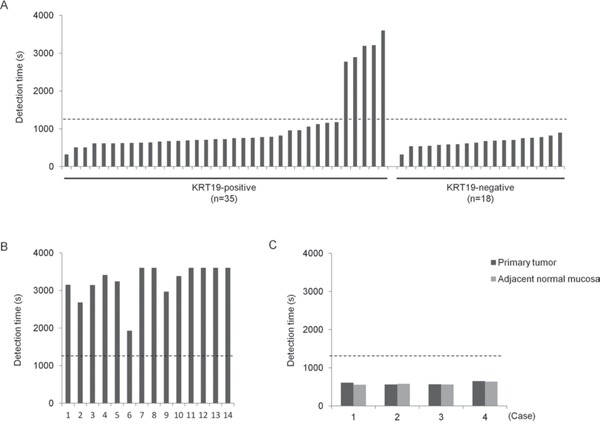
Detection of ANXA8 mRNA in lymph nodes and primary tumors from OSCC patients by RT-LAMP **A.** The expression of ANXA8 mRNA in KRT19-positive (n=35) or negative (n=18) metastatic lymph nodes. **B.** The expression of ANXA8 mRNA in normal lymph nodes (n=14) from non-cancer patients. **C.** The expression of ANXA8 mRNA in primary tumor and adjacent normal oral mucosa tissues from OSCC patients (n=4). The dotted lines indicate a cut-off time of 1,200 s (20 min).

To evaluate whether ANXA8 mRNA could be detected in metastatic lymph nodes by RT-LAMP, we designed 5 primer sets using PrimerExplorer and then determined which one amplified mRNA most efficiently, as described in the Methods section. We then carried out RT-LAMP assays using 133 lymph nodes, of which 53 were metastatic lymph nodes and 66 were non-metastatic lymph nodes from OSCC patients and 14 were normal lymph nodes from non-cancer patients. ANXA8 mRNA expression was detected rapidly in most metastatic lymph nodes but not in normal lymph nodes (Figure 4A, 4B). Using a cut-off time of 1,200 s (20 min), the sensitivity, specificity, and accuracy were 90.6% (48/53), 93.9% (62/66), and 92.4% (110/119), respectively (Table 2). RT-LAMP also detected ANXA8 in all KRT19-negative metastatic lymph nodes (Figure 4A). Furthermore, we examined the expression of ANXA8 mRNA in primary tumor and adjacent normal mucosa tissues from OSCC patients by RT-LAMP. ANXA8 mRNA was detected in both types of tissues at extremely high levels (Figure 4C).

**Table 2:** Detection rate of KRT19 and ANXA8 mRNA by RT-LAMP in lymph nodes classified as positive or negative for metastases histopathologically, using single or double markers

	KRT19	ANXA8	KRT19 + ANXA8
Histopathologically positive lymph nodes (n=53)	35 (3)^*^/53 (5) 66.0%	48 (5)/53 (5) 90.6%	53 (3)/53 (5) 100%
Histopathologically negative lymph nodes (n=66)	0 (0)/66 (33) 0%	4 (3)/66 (33) 6.1%	4 (3)/66 (33) 6.1%

( ), Number of sentinel lymph nodes

## DISCUSSION

In this study, we have identified ANXA8 and DSG3 as candidate molecular markers for molecular biology-based assays to detect metastases in whole lymph nodes from OSCC patients. DSG3 is also known as pemphigus vulgaris antigen (PVA) and is the calcium-binding, transmembrane glycoprotein component of desmosomes in vertebrate epithelial cells. DSG3 has been reported to be overexpressed in head and neck cancers and this has been correlated with lymph node metastasis and cell proliferation [19, 20]. Furthermore, several reports have already shown that DSG3 is a useful marker for detecting lymph node metastasis in these patients [21-23]. On the other hand, ANXA8 has been reported to be frequently highly overexpressed in pancreatic cancer [24], but little is known about its function and significance in other human cancers, including OSCC. Here, we present the first evidence that ANXA8 mRNA is commonly overexpressed in metastatic lymph nodes from OSCC patients.

Histopathological examination of serial sections and immunohistochemistry are useful for detecting metastases including micrometastases in the lymph nodes [25], but it is an expensive and challenging procedure for pathologists. We have therefore been using the RT-LAMP assay targeting KRT19 for SNB in OSCC, because this molecular biology-based test is easy, quick, and precise. However, we have seen several false negative cases due to low or no expression of KRT19 in primary OSCC tumors [17]. In this study, quite a few KRT19-negative metastatic lymph nodes were also observed, whereas we were able to detect ANXA8 mRNA in all those by qRT-PCR and RT-LAMP. Furthermore, ANXA8 mRNA was detected in a few lymph nodes in which metastases had not been identified histopathologically. These discordant results were due to tissue allocation bias. This suggests that molecular biology techniques may provide better diagnostic approaches than routine histopathological examination. These techniques could also be useful for detecting metastases in resected lymph nodes from neck dissections, when the number of metastatic lymph nodes in OSCC patients is used as an indication of the need for postoperative adjuvant therapy. However, the extracapsular spread as another indication of adjuvant therapy cannot be evaluated by these methods.

In summary, we have identified ANXA8 as a novel marker for detecting lymph node metastases in OSCC and have developed a molecular biology-based test using two markers, KRT19 and ANXA8 mRNAs. The accuracy, rapidity, and convenience of this system are superior to conventional histopathological examination, and should enhance our ability to precisely stage OSCC, with respect to “N” in the TNM status, and so improve the prognosis and quality of life of these patients.

## METHODS

### Samples from patients

All patients in this study were seen at the Ehime University Hospital between February 2007 and March 2011. The Ehime University Hospital Review Board approved the study and appropriate written informed consent was obtained from each patient. In the microarray analysis, we examined 7 metastatic lymph nodes from OSCC patients, 1 normal lymph node from a patient with ameloblastoma, and 5 salivary glands from patients with benign salivary gland diseases. In the qRT-PCR analysis, we examined 23 metastatic lymph nodes from OSCC patients and 9 normal lymph nodes from patients undergoing surgery for benign oral disorders. In the RT-LAMP assay, 392 lymph nodes resected by SNB or neck dissection from 103 patients with OSCC, 14 normal lymph nodes from 9 non-cancer patients, and 4 primary OSCC tissues were used. Each lymph node was rinsed quickly in saline solution to remove attached blood, and then divided into three portions. A central portion was examined through sectioning of the maximal cut surface of the specimen, stained with hematoxylin and eosin (H&E), and diagnosed as metastasis or non-metastasis. Remaining both sides were used for total RNA extraction.

### Total RNA extraction

For microarray and qRT-PCR assays, total RNA was extracted by lysing the tissues using ISOGEN (NipponGene, Tokyo, Japan), following the manufacturer's instructions, and homogenizing them in 0.5 ml ISOGEN using TissueLyser (Qiagen, Valencia, CA). RNA integrity was confirmed using the Agilent 2100 Bioanalyzer (Agilent Technologies, Santa Clara, CA, USA).

### Microarray analysis

The Applied Biosystems Chemiluminescent RT-IVT Labeling Kit (Life Technologies, Carlsbad, CA) was used to convert total RNA to digoxigenin (DIG)-labeled cRNA. Double-stranded cDNA was generated from 1 μg total RNA, transcribed using DIG-labeled nucleotides (Roche Diagnostics, Basel, Switzerland), fragmented, and hybridized to Human Genome Survey Arrays (Life Technologies) according to the manufacturer's instructions. After washing each array, the signal was developed using a chemiluminescent detection kit (Life Technologies). Processed arrays were scanned with a 1700 chemiluminescent microarray analyzer (Life Technologies) and the results analyzed using the GeneSpring GX 13.0 (Agilent Technologies). The raw microarray data have been deposited in the Gene Expression Omnibus (GEO, http://www.ncbi.nlm.nih.-gov/geo, experiment number: GSE70604), according to the minimum information about microarray experiment (MIAME) guidelines.

### qRT-PCR

We performed qRT-PCR in 10 μl final volumes in capillary tubes in a LightCycler instrument (Roche Diagnostic, Mannheim, Germany), using the One-Step SYBR PrimeScript RT-PCT Kit II (Takara Bio, Otsu, Japan). Reaction mixtures contained 5 μl 2 x One-Step SYBR RT-PCT Buffer 4, 0.4 μl PrimeScript One-Step Enzyme Mix 2, 0.4 μl of each primer, and 100 ng total RNA sample. PCRs were run for 40 cycles of 5 s at 95°C and 20 s at 60°C. At the end of each PCR run, melting curve analysis from 65 to 95°C was used to detect non-specific PCR products and primer-dimer co-amplification. Fluorescence curves were analyzed using LightCycler software version 3.5. The quantification of mRNA levels used the threshold cycle (CT). We used hydroxymethylbilane synthase (HMBS) expression as the endogenous control. The sequences of the primers used were as follows: KRT6C, forward 5′-CTG GAG GCA TCC AAG AGG TCA-3′ and reverse 5′-GCA GGG TCC ACT TTG TTT CCA-3′; ANXA8, forward 5′-CAG ACA CAA GTG GCT ACC TGG AGA-3′ and reverse 5′-TCC ACA AAG CTG CTC ACA TCA TC-3′; DSG3, forward 5′-CCC AGT TCC TGA TGG CTC AGA-3′ and reverse 5′-AAA TCG GCT CCA TTG GCT GTT A-3′; HMBS, forward 5′-CAT GCA GGC TAC CAT CCA TGT-3′ and reverse 5′-GTT AGC AGT GAT GCC TAC CAA-3′.

### RT-LAMP

We designed the primer sets for detecting ANXA8 mRNA expression using PrimerExplorer (http://primerexplorer.jp/). Each primer set consisted of 4 primers: a forward inner primer (FIP), F3, a backward inner primer (BIP), and B3. We selected 5 primer sets and evaluated each one for the time taken to detect the expression of ANXA8 mRNA, using a small number of lymph nodes. We used 2 metastatic lymph nodes and 1 normal lymph node, as defined histopathologically. We selected the primer set giving the fastest detection time and then designed a loop primer set. In RT-LAMP, adding loop primers to increase the starting points for DNA synthesis reduces detection times by one third to one half [26]. We also evaluated the detection times for 5 loop primer sets using a small number of lymph nodes, as described above. We were then able to identify the following primer set optimized for detecting ANXA8 mRNA: FIP 5′-TGC AGC TCC TTG GCT TCG TAT CTC TGA GCT CAG TGG CAA GT-3′ and BIP 5′-TGA CGC CAT GAA GGG CTT AGG GCT GGT TCT TGG TCC GAG-3′; loop-forward (LF) 5′-AGG GCC ACA ATG AGC CT-3′ and loop-backward (LB) 5′-CAA GGA GGG TGT CAT CAT TGA G-3′; F3 5′-GGA CCT CAC TGA GAC CTT GA-3′ and B3 5′-CGC CTT CAT TAT CTC CCG C-3′.

Each lymph node or tissue was homogenized in 4 ml of the homogenizing reagent Lynorhag, pH 3.5 (Sysmex, Kobe, Japan), for 90 seconds on ice using a Physcotron NS-310E2 microhomogenizer with an NS-4 microshaft (Microtec Nition, Funabashi, Japan) and centrifuged at 10,000 x g at room temperature. Two μl of the supernatant was subjected to the RT-LAMP. We used an RNA amplification kit for RT-LAMP and Loopamp reaction tubes (Eiken Chemical, Tokyo, Japan), each containing a 20 μl reaction mix, composed of 12.5 μl Reaction Mix, 40 pmol FIP and BIP, 20 pmol LF and LB primers, 5 pmol F3 and B3 primers, 1 μl Enzyme Mix, and 5 μl sample RNA. RT-LAMP assays were run at 65°C for 60 min and then the enzyme was inactivated by incubating the mixtures for 5 min at 80°C in Realoop-30 (Shoko Scientific, Yokohama, Japan). The expression level of mRNA was detected by real-time monitoring of turbidity changes caused by an increase in the magnesium pyrophosphate concentration, a by-product of the amplification reaction. The primer sequences for KRT19 mRNA were FIP 5′-GGA GTT CTC AAT GGT GGC ACC AAC TAC TAC ACG ACC ATC CA-3′, BIP 5′-GTC CTG CAG ATC GAC AAC GCC TCC GTC TC AAA CTT GGT TCG-3′, LF 5′-AGA ATC TTG TCC CGC AGG-3′, LB 5′-CGT CTG GCT GCA GAT GA-3′, F3 5′-TGG TAC CAG AAG CAG GGG-3′ and R3 5′-GTT GAT GTC GGC CTC CAC G-3′ [17].

### Statistical analysis

The student's t-test was used to analyze the distribution of CT values according to histological examination. The significance level was set at *p* < 0.05 for each analysis.

## References

[1] Jemal A, Bray F, Center MM, Ferlay J, Ward E, Forman D (2011). Global cancer statistics. CA: A Cancer Journal of Clinicians.

[2] Kreppel M, Eich HT, Kubler A, Zoller JE, Scheer M (2010). Prognostic value of the sixth edition of the UICC's TNM classification and stage grouping for oral cancer. Journal of Surgical Oncology.

[3] Kurokawa H, Zhang M, Matsumoto S, Yamashita Y, Tomoyose T, Tanaka T, Fukuyama H, Takahashi T (2005). The high prognostic value of the histologic grade at the deep invasive front of tongue squamous cell carcinoma. Journal of Oral Pathology & Medicine.

[4] Noguchi M, Kido Y, Kubota H, Kinjo H, Kohama G (1999). Prognostic factors and relative risk for survival in N1-3 oral squamous cell carcinoma: a multivariate analysis using Cox's hazard model. British Journal of Oral and Maxillofacial Surgery.

[5] Bajwa MS, McMillan R, Khattak O, Thomas M, Krishnan OP, Webster K (2011). Neck recurrence after level I-IV or I-III selective neck dissection in the management of the clinically N0 neck in patients with oral squamous cell carcinoma. Head & Neck.

[6] Grandi C, Alloisio M, Moglia D, Podrecca S, Sala L, Salvatori P, Molinari R (1985). Prognostic significance of lymphatic spread in head and neck carcinomas: therapeutic implications. Head & Neck Surgery.

[7] Stuckensen T, Kovacs AF, Adams S, Baum RP (2000). Staging of the neck in patients with oral cavity squamous cell carcinomas: a prospective comparison of PET, ultrasound, CT and MRI. Journal of Craniomaxillofacial Surgery.

[8] Yoon DY, Hwang HS, Chang SK, Rho YS, Ahn HY, Kim JH, Lee IJ (2009). CT, MR, US, 18F-FDG PET/CT, and their combined use for the assessment of cervical lymph node metastases in squamous cell carcinoma of the head and neck. European Radiology.

[9] D'Cruz AK, Vaish R, Kapre N, Dandekar M, Gupta S, Hawaldar R, Agarwal JP, Pantvaidya G, Chaukar D, Deshmukh A, Kane S, Arya S, Ghosh-Laskar S (2015). Elective versus therapeutic neck dissection in node-negative oral cancer. The New England Journal of Medicine.

[10] Coughlin A, Resto VA (2010). Oral cavity squamous cell carcinoma and the clinically n0 neck: the past, present, and future of sentinel lymph node biopsy. Current Oncology Reports.

[11] Hamakawa H, Onishi A, Sumida T, Terakado N, Hino S, Nakashiro K, Shintani S (2004). Intraoperative real-time genetic diagnosis for sentinel node navigation surgery. International Journal of Oral Maxillofacial Surgery.

[12] O'Connor R, Pezier T, Schilling C, McGurk M (2013). The relative cost of sentinel lymph node biopsy in early oral cancer. Journal of Cranio-Maxillofacial Surgery.

[13] Notomi T, Okayama H, Masubuchi H, Yonekawa T, Watanabe K, Amino N, Hase T (2000). Loop-mediated isothermal amplification of DNA. Nucleic Acids Research.

[14] Tamaki Y, Akiyama F, Iwase T, Kaneko T, Tsuda H, Sato K, Ueda S, Mano M, Masuda N, Takeda M, Tsujimoto M, Yoshidome K, Inaji H (2009). Molecular detection of lymph node metastases in breast cancer patients: results of a multicenter trial using the one-step nucleic acid amplification assay. Clinical Cancer Research.

[15] Güller U, Zettl A, Worni M, Langer I, Cabalzar-Wondberg D, Viehl CT, Demartines N, Zuber M (2012). Molecular investigation of lymph nodes in colon cancer patients using one-step nucleic acid amplification (OSNA): a new road to better staging?. Cancer.

[16] Hiraki S, Sakamoto N, Horio T, Kumano I, Otomo Y, Mochizuki H, Yamamoto J, Hase K (2011). One-step nucleic acid amplification (OSNA) for the application of sentinel node concept in gastric cancer. Annals of Surgical Oncology.

[17] Goda H, Nakashiro K, Oka R, Tanaka H, Wakisaka H, Hato N, Hyodo M, Hamakawa H (2012). One-step nucleic acid amplification for detecting lymph node metastasis of head and neck squamous cell carcinoma. Oral Oncology.

[18] Shinohara M, Harada T, Nakamura S, Oka M, Tashiro H (1992). Heterotopic salivary gland tissue in lymph nodes of the cervical region. International Journal of Oral Maxillofacial Surgery.

[19] Chen YJ, Chang JT, Lee L, Wang HM, Liao CT, Chiu CC, Chen PJ, Cheng AJ (2007). DSG3 is overexpressed in head and neck cancer and is a potential molecular target for inhibition of oncogenesis. Oncogene.

[20] Wang L, Liu T, Wang Y, Cao L, Nishioka M, Aguirre RL, Ishikawa A, Geng L, Okada N (2007). Altered expression of desmocollin 3, desmoglein 3, and β-catenin in oral squamous cell carcinoma: correlation with lymph node metastasis and cell proliferation. Virchows Archiv.

[21] Ferris RL, Xi L, Raja S, Hunt JL, Wang J, Gooding WE, Kelly L, Ching J, Luketich JD, Godfrey TE (2005). Molecular staging of cervical lymph nodes in squamous cell carcinoma of the head and neck. Cancer Research.

[22] Solassol J, Burcia V, Costes V, Lacombe J, Mange A, E Barbotte E, de Verbizier D, Cartier C, Makeieff M, Crampette L, Boulle N, Maudelonde T, Guerrier B, Garrel R (2010). Pemphigus vulgaris antigen mRNA quantification for the staging of sentinel lymph nodes in head and neck cancer. British Journal of Cancer.

[23] Ferris RL, Stefanika P, Xi L, Gooding W, Seethala RR, Godfrey TE (2012). Rapid molecular detection of metastatic head and neck squamous cell carcinoma as an intraoperative adjunct to sentinel lymph node biopsy. Laryngoscope.

[24] Karanjawala ZE, Illei PB, Ashfaq R, Infante JR, Murphy K, Pandey A, Schulick R, Winter J, Sharma R, Maitra A, Goggins M, Hruban RH (2008). New markers of pancreatic cancer identified through differential gene expression analyses: claudin 18 and annexin A8. American Journal of Surgical Pathology.

[25] Trivedi NP, Ravindran HK, Sundram S, Iyer S, Kekatpure V, Durah S, Kuriakose MA (2010). Pathologic evaluation of sentinel lymph nodes in oral squamous cell carcinoma. Head & Neck.

[26] Nagamine K, Hase T, Notomi T (2002). Accelerated reaction by loop-mediated isothermal amplification using loop primers. Molecular and Cellular Probes.

